# Knowledge Enhancement of Elementary School Staff about Traumatic Dental Injuries Using Different Educational Tools

**DOI:** 10.1155/2023/9167041

**Published:** 2023-12-06

**Authors:** Mojtaba Salehi Karizmeh, Fatemeh Mozaffari, Monirsadat Mirzadeh, Razieh Jabbarian

**Affiliations:** ^1^Department of Oral & Maxillofacial Surgery, Faculty of Dentistry, Tehran University of Medical Sciences, Tehran, Iran; ^2^Department of Oral & Maxillofacial Surgery, Research Center for Prevention of Oral and Dental Diseases, Baqiyatallah University of Medical Sciences, Tehran, Iran; ^3^Department of Community Medicine, Metabolic Diseases Research Center, Research Institute for Prevention of Non-Communicable Diseases, Qazvin University of Medical Sciences, Qazvin, Iran; ^4^Department of Pediatric Dentistry, Dental Caries Prevention Research Center, Qazvin University of Medical Sciences, Qazvin, Iran

## Abstract

**Introduction:**

This study aimed to assess the efficacy of different educational tools for knowledge enhancement of elementary school staff about the management of traumatic dental injuries (TDIs).

**Materials and Methods:**

This quasi-experimental (pretest–posttest) study was conducted on 126 elementary school staff in Qazvin city, who were randomly selected by the cluster sampling in 2020. The baseline knowledge level of the participants about TDIs was assessed by an online questionnaire. Next, they were randomized into the poster, video clip, and no-intervention control groups (56 samples in each group). An informatory poster and a video clip about TDIs were sent to the participants in the first two groups, respectively, through the WhatsApp instant messaging app, and the knowledge level of the three groups was assessed again after 3 weeks. Data were analyzed by ANOVA, McNemar test, chi-square test, and *t*-test.

**Results:**

A significant correlation was noted between the baseline knowledge level of the participants and their educational level, participation in first aid courses covering TDIs, and history of encountering TDIs (*P* < 0.05). The knowledge level of the participants significantly increased after the intervention in the poster and video clip groups (*P* < 0.05). Knowledge enhancement was 43.26% in the poster and 36.61% in the video clip group (*P* > 0.05).

**Conclusion:**

Despite the low-baseline knowledge level of the elementary school staff in Qazvin city about TDIs, their knowledge level significantly improved after the educational interventions.

## 1. Introduction

Traumatic dental injuries (TDIs) can significantly compromise the oral health of children and may bring about esthetic, functional, and psychological consequences for them [1]. They can adversely affect the self-esteem of children as well [2]. According to the literature, over 20% of school-aged children experience TDIs [3]. Schools are the most common location of occurrence of TDIs in children over 7 years of age, and the majority of them is sport injuries [2]. The prognosis of treatment of TDIs, the most important of which being avulsion, depends on their immediate emergency management [3]. Thus, the role of school staff in the management of TDIs is undeniable, and enhancement of the public knowledge about the management of TDIs is imperative [4]. Availability of emergency services and public knowledge regarding how to access or request such services are also important [2].

Considering the high frequency of occurrence of TDIs in schools, the school staff plays an important role in the emergency management of TDIs because they are often the first individuals at the scene [1]. A previous study regarding the knowledge level of teachers about the management of TDIs pointed to their low-knowledge level such that many teachers believed that an avulsed tooth cannot be replanted [5]. Raoof et al. [4] found that most of their participants (94.3%) were dissatisfied with their insufficient level of knowledge about tooth and alveolar fracture management [4]. Researchers have recommended continuing education courses for teachers regarding dental trauma as a good action plan [1]. Educational pamphlets have also been recognized as a suitable and efficient method to raise awareness and improve the knowledge of teachers in this regard [3].

In light of the current health societal conditions, it seems that online educational interventions are becoming increasingly necessary. Despite all previous efforts to improve the awareness of teachers and school staff, there are few studies that have used electronic methods. Considering this and the significance of early management of TDIs, particularly avulsion, the significant role of the school staff in this respect, and their reportedly low-knowledge level about the management of TDIs, this study aimed to assess the efficacy of different educational tools for knowledge enhancement of elementary school staff in Qazvin city regarding the management of TDIs.

## 2. Materials and Methods

This quasi-experimental (pretest–posttest) study was conducted on the elementary school staff including the personnel of the executive office, teachers, and trainers in Qazvin city.

**Figure 1:**
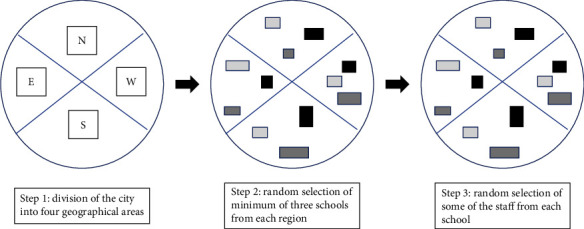
Cluster sampling.

After obtaining permission from the Ministry of Education and Training, classified random sampling was performed in two phases. First, a list of all elementary schools in Qazvin city was obtained, and the city was divided into four geographical locations of north, south, west, and east. By cluster random sampling, a minimum of three schools were randomly selected from each of the four geographical regions, and some of the staff from each school were randomly selected proportionate to the population, such that eventually, an equal number of personnel were selected from each of the four geographical regions (both males and females) (Figure 1). According to a study by Ghadimi et al. [6], the sample size was calculated to be 105:

(1) $$\begin{array}{c} n=\frac{{\left(Z_{1-a/2}\right)}^{2}p_{q}}{d^{2}}, \end{array}$$

where alfa=0.05, *d*=0.07, *p*=0.67, *Z*_1−*a*/2_=1.961150826, and *n*=105.

To increase the accuracy of the results, 168 individuals were enrolled; out of which, 126 returned the questionnaires and their data were statistically analyzed. The participants were divided into three groups. Each group comprised 56 executive staff, school teachers, and trainers. An online questionnaire was first used to assess the baseline knowledge level of the participants regarding the management of TDIs [6]. Next, WhatsApp instant messaging app was used to send the poster file to the first group and a 2-min educational video clip to the second group. The third group served as the control group and did not receive any educational intervention. After 3 weeks, the participants in all three groups were asked to fill out the online questionnaire again. The difference in the number of participants' correct answers was considered as the degree of change in their knowledge level. The questionnaire included 18 questions in three domains as follows, which included more common TDIs and most important TDI management strategies [6].

The first domain included 11 questions regarding the age, gender, level of education, work experience, previous participation in first aid courses, time of participation in first aid courses, coverage of TDIs in the first aid course, field of education related to medical fields, having a school-aged child, history of exposure to TDIs, and referral location of children in case of TDIs.

The second domain included three questions regarding three different scenarios of TDIs including crown fracture, laceration, and avulsion.

The third domain included four questions regarding important points about cleaning of avulsed teeth, referral of patients with TDIs, and the medium for storage and transfer of avulsed teeth [6].

The educational clip covered the management of TDIs in 2 min. The clip explained the different types of TDIs that may occur at school and the role of elementary school staff in emergency management of TDIs such as fracture, luxation, and avulsion, and proper management of such conditions. The poster included instructions regarding diagnosis and management of three types of TDIs namely crown fracture, luxation, and avulsion in the form of text and image.

Data were analyzed using SPSS version 25 (IBM Corp. Armonk, NY) by the McNemar test, ANOVA, chi-square test, and *t*-test. Level of significance was set at 0.05.

## 3. Results

A total of 168 individuals participated in this study; out of which, 41 (24.4%) did not fill out the second questionnaire and were excluded.  Table 1 presents the demographic information of the participants. As shown, females, those in the age range of 40–49 years, and those with 5–10 years of work experience had a higher frequency in all three groups. The chi-square test found no significant difference among the three groups in gender, age, or work experience of the personnel (*P* > 0.05). The number of personnel who had school-aged children was higher than the number of those who did not (*P*=0.002).

Table 2 presents the history of participation of the personnel in the first aid courses. The number of personnel who had not participated in a first aid course was higher than the number of those who had participated in such courses in all three groups, but not significantly (*P*=0.813). However, significant differences were noted in time of participation in such courses and coverage of TDIs in the course.

Regarding the level of education, the majority of the participants had a Bachelor's degree (*P*=0.043) and the field of education of the majority of them was not related to medical fields (*P*=0.502). Also, the majority of them had not ever encountered TDIs (*P*=0.936). The three groups were not significantly different regarding the abovementioned parameters.

Of all participants, 34 had encountered TDIs; out of which, the adopted management was correct by 10 (29.41%), and incorrect by 21 (61.76%) participants; 3 (8.82%) did not do anything. Also, regarding the location of referral of children with avulsion, 38% reported taking them to a pediatric dentist, 36% reported taking them to a general dentist, 16% reported taking them to a dental school clinic, 7% reported taking them to the emergency department of hospitals, and 3% reported taking them to a physician's office.

A significant correlation existed between the level of education of the personnel and their baseline (before the intervention) knowledge level about TDIs (*P*=0.009), and the knowledge level was significantly higher in those with a Master's degree or higher. Moreover, the baseline knowledge level of the personnel who had passed a first aid course covering TDIs was significantly higher than that of others (*P* < 0.05). A significant correlation was also noted between the history of encountering TDIs and baseline knowledge level of the personnel (*P*=0.020).

Table 3 presents the knowledge score of the participants regarding different domains of management of TDIs before and after the intervention. The results indicated that the percentage of correct answers after the educational intervention in the poster group was higher than that before the intervention, and the improvement in all domains was significant in the poster group (McNemar test, *P* < 0.05). The percentage of correct answers after the educational intervention in the video clip group was higher than that before the intervention, and the improvement in the management of crown fracture, management of avulsion, management of luxation, knowledge about measures to be taken in case of avulsion, and knowledge about the medium for avulsed teeth was significant (McNemar test, *P* < 0.05). In the control group, the improvement in management of crown fracture and knowledge about measures to be taken in case of avulsion was significant (*P* < 0.05). No other significant change was noted.

Table 4 compares the knowledge level of the three groups regarding different domains. The maximum improvement was noted in the poster group (43.26% improvement) followed by the video clip group (36.61%). Also, the maximum improvement in the poster group was related to knowledge about the suitable medium for avulsed teeth (57%), and management of crown fracture (53.85%) while the maximum improvement in the video clip group occurred in knowledge level about the suitable medium for avulsed teeth (53%). According to the chi-square test, a significant difference was noted in management of crown fracture and luxation between the poster and video clip groups both before and after the intervention (*P* < 0.05). However, the difference in knowledge level was not significant between the video clip and poster groups before or after the intervention (*P* > 0.05).

Linear regression analysis was implemented to examine the effect of baseline parameters on the degree of knowledge gain; results showed that the only factor affecting the outcome was the study group (*P* < 0.001) with the highest impact belonging to the poster group. None of the other factors had a significant impact (*P* > 0.05) (Table 5).

## 4. Discussion

Knowledge about the emergency management of TDIs at the site is highly important. Considering the high prevalence of TDIs in school-aged children and reportedly low-knowledge level of the school staff in this respect [6, 7], this study aimed to assess the efficacy of an educational video clip and a poster for knowledge enhancement of elementary school staff about TDIs. Teachers, trainers, and the executive staff of the elementary schools were the target population of this study, who were mainly female, had a Bachelor's degree, and were between 40 and 49 years. After the educational intervention, the knowledge level improved by 43.26% in the poster group and by 36.61% in the video clip group. In both groups, maximum improvement occurred in knowledge about the best medium for storage of avulsed teeth (57% in the poster and 53% in the video clip group). The baseline knowledge level of the personnel was significantly correlated with their educational level, participation in first aid courses covering TDIs, and history of encountering TDIs, which highlights the role of education (theoretical and practical) in knowledge enhancement. Also, encountering a case of TDI urges the person to seek information in this respect and results in knowledge enhancement, and probably improves the performance on next encounters. Kamali et al. [8] found no significant correlation between the knowledge score of school health instructors and their age or work experience; however, those who had received training in this regard had a significantly higher knowledge level than those without such training. Ivanda et al. [7] found that previous exposure to traumatic dental injuries significantly improved the teachers' knowledge about the management of TDIs but in contrast to our findings, they did not find any correlation with academic or professional education. Also, Feldens et al. [9] reported lower knowledge level of teachers with less experience, those who had not participated in first aid courses, and those who had no prior encounter with TDIs.

The content presented in both poster and clip methods was the same and based on the recommendations of the latest guidelines of the International Association of Dental Traumatology [10]. In this guideline, recommendations regarding the emergency management of permanent tooth avulsion at the site of the accident are presented in eight steps, on which all general recommendations are based [10].

Due to the COVID-19 pandemic, this study was conducted by using an online questionnaire, and the interventions were also performed online by sending the poster and video clip files to the participants via the WhatsApp instant messaging app. E-health is a new concept which has caught the interest of the World Health Organization. It refers to the use of Internet, social media, and mobile technology for health purposes [2]. The present results confirmed the optimal efficacy of online educational interventions. Most school teachers in the study by Altamimi et al. [11] preferred e-learning via smartphones, while lectures and printed brochures were the least popular routes of receiving information. Gaffar et al. [12] recommended the use of social media in order to share TDI educational content and motivate teachers about learning.

The efficacy of multimedia educational interventions is expected to be higher than online posters. However, in the present study, the online poster was slightly more effective than the video clip in knowledge enhancement (43% versus 36%); although this difference did not reach statistical significance. This finding may be attributed to the online conduction of educational interventions because playing a video clip requires downloading the file first and having adequate Internet speed in order to watch it with no interruption. Also, the information can be acquired from the online poster by a short glance while the video clip takes 2 min to watch. However, further studies are required on the efficacy of shorter online educational interventions compared with multimedia.

Nashine et al. [13] evaluated the knowledge and attitude of 158 school teachers before and after an educational lecture as a classic method of training by using a questionnaire and reported significant improvement in their knowledge and attitude after the intervention (from 0.6% to 56.3%). Taranath et al. [14] assessed the knowledge and attitude of 214 elementary school teachers about the emergency management of traumatized teeth and evaluated the efficacy of PowerPoint presentation for knowledge enhancement by using a questionnaire. They reported significant improvement of knowledge and attitude of students in this respect from 0 at baseline to 70.83% after the intervention. Ghadimi et al. [6] assessed the efficacy of educational posters in elementary schools for knowledge enhancement of 40 health instructors about the management of TDIs. After the intervention, 93.3% of the test group versus 60% of the controls correctly responded to the questions regarding the management of crown fracture; these rates were 63.3% versus 40%, respectively, regarding luxation. Al-Asfour et al. [15] evaluated the knowledge level of teachers about avulsion and dental emergencies before and after an educational intervention by a lecture and showed that the knowledge level of teachers about avulsion increased from 39% to 97% after a 30-min lecture in this regard.

The present results also indicated the positive efficacy of both poster and video clip for knowledge enhancement of the school staff. However, lower level of knowledge enhancement in this study (40%) compared with previous investigations may be related to the higher efficiency of traditional classroom-based instruction. In general, online health education requires designing specific platforms with visual attractions to achieve maximum efficiency.

This study had some limitations, including legal issues related to the implementation of the study in schools and the impossibility of obtaining a practical feedback from the study participants. Further studies on a larger sample size are required to compare the efficacy of traditional and online instruction for knowledge enhancement of personnel of different age groups regarding TDIs to find the method with maximum cost-effectiveness. Also, future studies should assess the participants' performance in the simulated clinical scenarios.

## 5. Conclusion

Higher educational level, participation in first aid courses covering TDIs, and history of encountering TDIs were significantly correlated with higher baseline knowledge about TDIs. Despite the low-baseline knowledge level of the elementary school staff in Qazvin city about TDIs, their knowledge level significantly increased after the educational interventions in the form of poster and video clip. Knowledge enhancement was 43.26% in the poster and 36.61% in the video clip group. When considering all parameters together on regression analysis, the study group was the only factor to affect the outcome of knowledge enhancement.

## Figures and Tables

**Table 1:** Demographic information of the participants.

Parameter	Categories	Groups			*P*-value
		Video clip	Poster	Control	
Gender	Female	26 (31.0%)	27 (32.1%)	31 (36.9%)	0.725
	Male	16 (38.1%)	12 (28.6%)	14 (33.3%)	

Age groups (yr)	20–29	8 (22.2%)	14 (38.9%)	14 (38.9%)	0.492
	30–39	10 (28.6%)	11 (31.4%)	14 (40.0%)	
	40–49	17 (44.7%)	9 (23.7%)	12 (31.6%)	
	>50	7 (41.2%)	5 (29.4%)	5 (29.4%)	

Work experience (yr)	<1	6 (30.0%)	9 (45.0%)	5 (25.0%)	0.051
	1–5	6 (35.3%)	5 (29.4%)	6 (35.3%)	
	5–10	4 (12.9%)	14 (45.2%)	13 (41.9%)	
	10–15	15 (50.0%)	3 (10.0%)	12 (40.0%)	
	>15	11 (39.3%)	8 (28.6%)	9 (32.1%)	

**Table 2:** History of participation in first aid courses by the personnel.

Parameter	Category	Groups			*P*-value
		Video clip	Poster	Control	
History of participation in first aid course	Yes	16 (34.0%)	13 (27.7%)	18 (38.3%)	0.813
	No	26 (32.9%)	26 (32.9%)	27 (34.2%)	

When did you participate in the first aid course?	I did not	12 (20.0%)	22 (36.7%)	26 (43.3%)	0.008
	<1 year ago	6 (42.9%)	8 (57.1%)	0 (.0%)	
	1–5 years ago	5 (35.7%)	4 (28.6%)	5 (35.7%)	
	5–10 years ago	10 (52.6%)	3 (15.8%)	6 (31.6%)	
	10–15 years ago	9 (47.4%)	2 (10.5%)	8 (42.1%)	

Did the course cover TDIs?	I did not participate in any course	16 (22.2%)	27 (37.5%)	29 (40.3%)	0.009
	Yes	13 (65.0%)	3 (15.0%)	4 (20.0%)	
	No	13 (38.2%)	9 (26.5%)	12 (35.3%)	

**Table 3:** Knowledge score of the participants regarding different domains of management of TDIs before and after the intervention.

Variable	Poster group			Video clip group			Control group		
	Before (%)	After (%)	*P*	Before (%)	After (%)	*P*	Before (%)	After (%)	*P*
Management of crown fracture	30.77	84.62	≤0.001	38.10	71.43	0.001	20.00	48.89	0.011
Management of avulsion	12.82	58.97	≤0.001	40.48	66.67	0.035	35.56	24.44	0.383
Management of luxation	23.08	48.72	0.013	14.29	64.29	≤0.001	13.33	15.56	1.000
Knowledge about emergency management of avulsion	58.97	84.62	0.006	50.00	83.33	0.077	91.11	62.22	0.791
Knowledge about measures to be taken in case of avulsion	20.51	71.79	0.021	33.33	57.14	0.001	15.56	15.56	0.002
Knowledge about the medium for avulsed teeth	10	67	≤0.001	26	79	0.041	9	2	1.000

**Table 4:** Comparison of the knowledge level of the three groups regarding different domains.

Variable	Groups			*P*-value
	Video clip	Poster	Control	
Management of tooth fracture	33.33	53.85	28.89	0.016
Management of avulsion	26.19	46.15	−11.12	0.325
Management of luxation	50	25.64	2.23	0.012
Knowledge about emergency management of avulsion	33.33	25.65	−28.89	0.416
Knowledge about measures to be taken in case of avulsion	23.81	51.28	0	0.810
Knowledge about the medium for avulsed teeth	53	57	−7	0.147
Mean percentage of change	36.61	43.26	−2.64	0.524

**Table 5:** Linear regression analysis for prediction of knowledge post intervention score.

	Unstandardized coefficients	Standardized coefficients	
	*B*	*B*	*P*-value
Study group	−1.15	−0.59	0.001

## Data Availability

The authors confirm that the data supporting the findings of this study are available within the article.

## Abbreviations

TDIs: Traumatic dental injuries
ANOVA: Analysis of variance.

## Supplementary Materials

Supplementary Materials Questionnaire used in the study.
