# Supplementary material for: Knowledge Enhancement of Elementary School Staff about Traumatic Dental Injuries Using Different Educational Tools
Source: Int J Dent. 2023 Dec 6;2023:9167041. doi: 10.1155/2023/9167041 (PMC10719041; doi:10.1155/2023/9167041)
Supplement: Supplementary Materials — Questionnaire used in the study. [file 9167041.f1.docx]

***Please read the following questions carefully and mark the correct option with an X.***

***This questionnaire has only a research aspect and participation in it is optional.***

***The questionnaire does not have names and the details of people are not available to anyone other than the researcher.***

***If there is an incomprehensible question, write the necessary explanation in front of it.***

**Demographic characteristics**

1-Gender: female male

2-Age: 20-29 30-39 40-49 50 and above

3- Degree of Education: Diploma Bachelor’s Degree Master’s Degree

4- How many years have you been working?

Under 1 year 1-5 years 5-10 years 10-15 years over 15 years

5- Have you ever completed a first aid course? yes no

6- If your answer is yes, how many years ago did you take this course?

Under 1 year 1-5 years 5-10 years 10-15 years over 15 years

7- Did your training course include injuries caused by dental trauma? Yes no

8- Have you ever had a close encounter with trauma and dental injuries? yes no

9- If your answer is positive, what action have you taken? ......

10-Do you have education related to medical sciences? yes no

11-Do you have a school-age child or children? yes no

**Performance questions**

12- A 9-year-old boy broke a part of his front tooth crown of upper jaw when a basketball hit his face while playing. What do you do in this case?

a) After school, I call the child's parents to explain the incident to them

b) I give the child a warm drink and call his parents

c) I am looking for a broken piece

d) I will take him to the dentist immediately

e) I call his parents to take the child to the dentist

13- An 8-year-old girl fell in the school yard and her front tooth fell out of her mouth and she is bleeding in her mouth. What is your action?

A) To control the bleeding, I put a gauze in the child's mouth at the bleeding site and do not do anything else

b) I find the fallen tooth and wash it well and I give the child to take home

c) I find the tooth and place it in the child's mouth and take it to the dentist.

d) I put the tooth in a liquid and quickly take the child to the dentist

e) I do not look for a fallen tooth and immediately refer the child to the dentist

14- A 10-year-old student is bleeding from the side of his tooth after hitting his face, but the crown of the tooth is healthy. What do you do for the child?

a) I give the child a warm drink and call his parents

b) I put a gauze on the bleeding site to control the bleeding

c) I call the child's parents to take him to the dentist

d) I will take him to the dentist immediately

e) Considering that the fracture did not occur, there is no need for any treatment

**Awareness questions**

15- If a child's tooth has been knocked out after an accident, Where should the child go for treatment?

A) Doctor

b) General dentist

c) Children's dentist

d) Hospital emergency department

e) Faculty of Dentistry

16- If a child's tooth has been knocked out after an accident, How long after the accident should the child go to the medical center?

a) immediately b) a few hours later c) a day later

d) after removal of facial swelling e) there is no need to visit

17- If the tooth has fallen on the ground after knocking out, what is the most suitable action?

a) The tooth should be cleaned with a toothbrush and handed over to the child's parents

b) The tooth should be washed with disinfectant or soap and handed over to the child's parents

c) The surface of the tooth should be cleaned with soap and water, then the tooth should be placed in its cavity in the child's mouth

d) The surface of the tooth should be gently washed with tap water and then placed in its cavity in the child's mouth

e) Without any other action, the tooth should be placed directly inside the child's mouth in its cavity

18- If the extracted tooth is to be kept in a material, which material is better?

A) paper towels or gauze

b) Fresh milk

c) Physiological serum

d) Disinfectant or alcohol

e) Ice
